# Intra-Articular Synovial Sarcomas: Incidence and Differentiating Features from Localized Pigmented Villonodular Synovitis

**DOI:** 10.1155/2015/903873

**Published:** 2015-12-24

**Authors:** D. Nordemar, J. Öberg, O. Brosjö, M. Skorpil

**Affiliations:** ^1^Department of Radiology, Capio S:t Göran Hospital, S:t Göransplan 1, 112 81 Stockholm, Sweden; ^2^Department of Medical Physics, Karolinska University Hospital, Karolinska Vägen 1, 171 76 Solna, Sweden; ^3^Department of Orthopaedics, Karolinska University Hospital, Karolinska Vägen 1, 171 76 Solna, Sweden; ^4^Department of Radiology, Karolinska University Hospital, Karolinska Vägen 1, 171 76 Solna, Sweden; ^5^Department of Molecular Medicine and Surgery, Karolinska Institute, Solnavägen 1, 171 77 Solna, Sweden

## Abstract

*Purpose.* To determine the incidence of intra-articular synovial sarcomas and investigate if any radiological variables can differentiate them from localized (unifocal) pigmented villonodular synovitis (PVNS) and if multivariate data analysis could be used as a complementary clinical tool.* Methods.* Magnetic resonance images and radiographs of 7 cases of intra-articular synovial sarcomas and 14 cases of localized PVNS were blindedly reviewed. Variables analyzed were size, extra-articular growth, tumor border, blooming, calcification, contrast media enhancement, effusion, bowl of grapes sign, triple signal intensity sign, synovial low signal intensity, synovitis, age, and gender. Univariate and multivariate data analysis, the method of partial least squares-discriminant analysis (PLS-DA), were used. Register data on all synovial sarcomas were extracted for comparison.* Results.* The incidence of intra-articular synovial sarcomas was 3%. PLS-DA showed that age, effusion, size, and gender were the most important factors for discrimination between sarcomas and localized PVNS. No sarcomas were misclassified as PVNS with PLS-DA, while some PVNS were misclassified as sarcomas.* Conclusions.* The most important variables in differentiating intra-articular sarcomas from localized PVNS were age, effusion, size, and gender. Multivariate data analysis can be helpful as additive information to avoid a biopsy, if the tumor is classified as most likely being PVNS.

## 1. Introduction

Magnetic resonance imaging (MRI) is the method of choice when examining soft tissue tumors [1, 2]. Some benign tumors, such as lipomas and hemangiomas, can be safely diagnosed using MRI without the need of a biopsy [1, 3, 4]. Intra-articular sarcomas however pose a diagnostic problem, since they have similar MRI features to benign localized (unifocal) pigmented villonodular synovitis (PVNS) [5–9]. Since asymptomatic localized PVNS does not require surgical intervention, avoiding unnecessary biopsies of the much more common localized PVNS, without missing sarcomas, would be valuable. Localized PVNS is a benign neoplastic process representing approximately 6% of all PVNS and can, if the lesion is symptomatic, be removed arthroscopically with a negligible risk of recurrence [9, 10]. Sarcomas on the other hand are intermediate or high-grade malignant tumors with a high potential for metastases. Extensive surgery is required, sometimes ending in amputation [8]. Synovial sarcomas have the highest incidence among intra-articular sarcomas [11]. The term “synovial” is a misnomer, as the tumor does not origin from synovia. It merely reflects the histopathological similarities to developing synovial tissue [5]. No radiological distinguishing features have been established to differentiate intra-articular synovial sarcomas from benign tumors and in the literature there are only case reports or reviews of case reports [5, 6, 12–16]. In this study we reviewed 7 patients with intra-articular synovial sarcomas, which is a comparatively large material of these rare tumors. These were blindly reviewed together with localized PVNS to evaluate if any variables would be useful for differentiation. We also investigated if multivariate data analysis could add differential diagnostic information. Finally, data from the Scandinavian Sarcoma Group (SSG) on synovial sarcomas were compared to the intra-articular synovial sarcomas.

## 2. Materials and Methods

### 2.1. Patients and Register Data

Within the SSG 7 cases (6 males and 1 female) of intra-articular synovial sarcomas have been recorded between the years 2000 and 2013, all having MRI examinations. The mean age was 21 years (range 9–35 years). Six were located in the knee joint and 1 in the elbow joint. All 7 cases had chronic pain and pain on movement. One patient had symptoms for 5 months and the other 6 for at least 1 year. All diagnoses were histopathologically confirmed by pathologists subspecialized in sarcomas, based on histological appearance in combination with immunohistochemistry. Four cases were also tested and found to be positive for the translocation between chromosome X and chromosome 18 (SYT-SSX), which is specific for synovial sarcomas [14]. Three synovial sarcomas were monophasic and four were biphasic. Four patients were treated by operation and chemotherapy, one patient had operation and radiotherapy, one patient had only operation, and one patient refused operation but was treated with both chemotherapy and radiotherapy. No metastases were found at presentation. One patient had a recurrence and died 2.5 years after diagnosis, while 6 patients are disease-free.

For comparison, 14 MRI examinations of patients with localized (unifocal) PVNS (5 males and 9 females) diagnosed at a Sarcoma Center were included. Mean age was 42 years (range 15–70 years). Seven cases had histopathological specimens and all were diagnosed by pathologists subspecialized in sarcomas and soft tissue pathology. The other 7 were not operated on, but all were followed up clinically for up to 3 years with no signs of progression.

A senior radiologist with more than 30 years of experience in bone and soft tissue tumors, blinded to the diagnoses and clinical data, reviewed all 21 MRI examinations with regard to variables chosen from previous studies [5, 17, 18]: largest diameter (size), extra-articular growth, tumor border (well-defined or infiltrative), bowl of grapes sign, triple signal intensity sign, blooming (magnetic susceptibility artifact seen on gradient echo sequences (GRE)), calcification on radiographs, and contrast media enhancement [8, 18]. The amount of effusion (“small” refers to normal amounts of fluid, “large” refers to the bursa suprapatellaris or elbow joint being clearly distended with fluid, and “moderate” refers to being in-between small and large), low signal intensity in the synovia (suggesting hemosiderin), and synovitis (general synovial contrast enhancement and/or “rice bodies”) were also investigated. MRI sequences differed since most cases were referred.

Data on soft tissue sarcomas were extracted from the SSG Central Register, where data on all sarcoma patients in Scandinavia from 1979 and forward are recorded. Up until July 2014, a total of 9327 soft tissue sarcomas were registered. 334 of them lacked some relevant information and were therefore excluded. A total of 446 cases of synovial sarcomas (Table 1) were diagnosed between 1986 and 2013, which amounts to 5% of all soft tissue sarcomas in the register. Mean age at diagnosis was 39 years (range 6–86 years). The male : female ratio was 1 : 1. Between 2000 and 2013 a total of 226 synovial sarcomas were diagnosed. During this period approximately 3% of the synovial sarcomas were intra-articular (7/226). In addition to the 7 synovial sarcomas, there was one case of an intra-articular chondrosarcoma and one case of an intra-articular liposarcoma in the SSG Register between 2000 and 2013.

### 2.2. Statistics

For univariate data analysis Kruskal-Wallis 1-way ANOVA and Fisher's exact test were used. Bonferroni correction was subsequently used. Phi was used for covariation analysis. *p* < 0.05 was chosen as the significance level.

For multivariate data analysis the method partial least squares-discriminant analysis (PLS-DA) was used (SIMCA; Umetrics AB, Umea, Sweden). PLS-DA relates data matrices to each other by a linear multivariate model. Before performing PLS-DA, data are mean-centered and scaled to unit variance. A *Y*-matrix is formed, encoding class membership by a set of “dummy” variables (e.g., group one = 0 and group two = 1). PLS-DA then relates the *X*-matrix, containing the observed data, and *Y* to each other by a linear multivariate model. The aim of PLS-DA is to create a predictive model that, using linear combinations of the variables, best separates groups within the data. The prediction parameter, *Q* ^2^, provides an estimate of the predictive power of a principal component. For the models built in this study *Q* ^2^ needs to be larger than 0.05. PLS-DA gives one or more vectors of scores (*t*), which summarizes all the variables entering the analysis. A score plot can be seen as a window in the *X*-space, displaying the observations (i.e., patients) as situated on the projection planes. The variable influence on projection (VIP) parameters reflect the importance of terms in the model with respect to both *Y* and *X*. Terms with large VIP, larger than 1, are the most relevant for explaining *Y*. Effusion was treated as a quantitative variable ranging from 1 (small) to 3 (large).

When analyzing SSG-data on all synovial sarcomas, receiver operating characteristics (ROC) analysis was used to find the best cut-off values for age and tumor size as prognostic factors.

## 3. Results

All patients with available MRI sequences had the same results for well-defined borders (all), bowl of grapes sign (none), triple signal intensity sign (none), and contrast enhancement (all), and these variables were excluded in the final analysis. Covariation was tested for size and calcification in the intra-articular sarcomas and was not significant (*p* = 0.06).

### 3.1. Univariate Data Analysis

Data for localized PVNS and intra-articular sarcomas are shown in Table 2. No variables were significant after Bonferroni correction.

### 3.2. Multivariate Data Analysis

**Figure 1:**
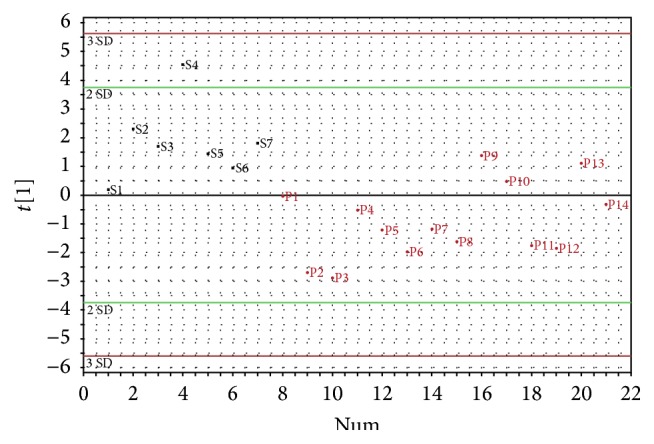
One-dimensional PLS-DA score plot showing the separation of the two groups. The color of each dot represents the actual diagnosis of that case, where black corresponds to sarcoma and red to localized PVNS. Green and red horizontal lines correspond to two or three standard deviations (SD) of the *t*_1_ vector.

**Figure 2:**
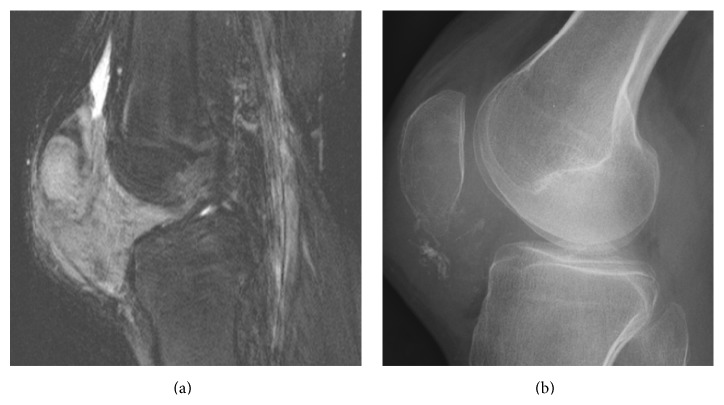
17-year-old boy with a large sarcoma in Hoffa's fat pad (case #S4). (a) Sagittal fat-saturated T2-weighted MR image shows the tumor growing extra-articularly and invading the patella. (b) Lateral radiograph shows intratumoral calcifications.

**Figure 3:**
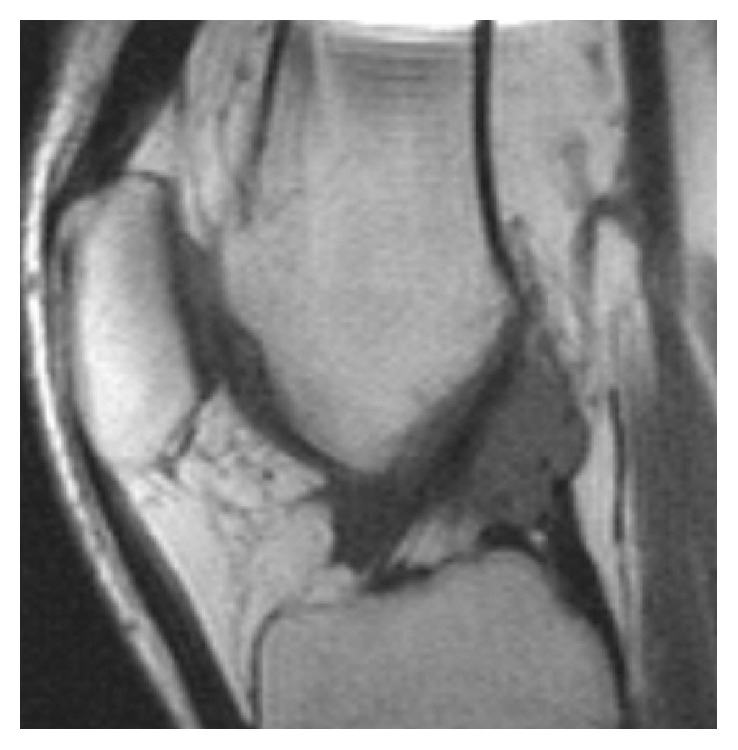
34-year-old man with a small sarcoma at the cruciate ligaments (case #S6). Sagittal T1-weighted MR image.

**Figure 4:**
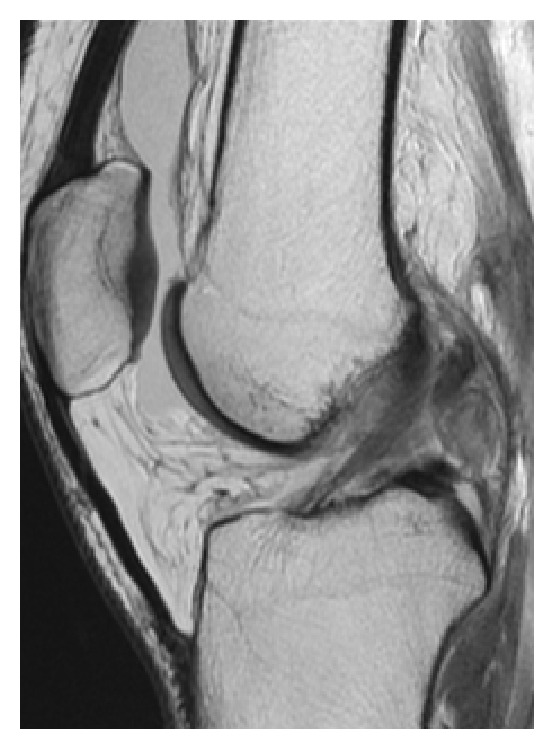
24-year-old female with localized PVNS at the posterior cruciate ligament (case #P5). Bursa suprapatellaris is clearly distended with fluid. Sagittal proton density-weighted MR image.

Calcification and blooming were excluded in the multivariate data analysis due to the number of cases with missing radiographs (12 cases) or missing GRE sequences (14 cases). PLS-DA gave a one-component model. *R* ^2^ *X*, the variance in *X* explained by the model, was 0.33. *R* ^2^ *Y*, the variance in *Y* explained by the model, was 0.51. *Q* ^2^ was 0.43.  Figure 1 shows the score plot of the significant component (*t*_1_) of the PLS-DA model. Figures 2 and 3 show two sarcomas with different *t*_1_ values: one with a high *t*_1_ and one with more indeterminate *t*_1_.  Figure 4 shows localized PVNS with large amounts of effusion.

The VIP of the PLS-DA in Figure 1 is shown in Table 3. VIPs are sorted in descending order of importance and it can be concluded that the variables age, effusion, size, and gender were the most important variables for the separation.

### 3.3. *ROC* Analysis

Most important prognostic factors on all synovial sarcomas were age ≤ 20 years and size ≤ 5 cm. 82% of patients aged ≤ 20 and 75% of patients with a tumor ≤ 5 cm had disease-free survival at latest follow-up.

## 4. Discussion

Using multivariate data analysis, the most important variables to differentiate intra-articular synovial sarcomas from localized PVNS were size, effusion, age, and gender. With univariate analysis no variables were significant after Bonferroni correction.

Differentiating intra-articular synovial sarcomas from benign tumors is difficult and no certain radiological features have been established. The most challenging differential diagnosis is localized PVNS [5, 6, 10, 12–16]. Using multivariate data analysis this study showed that the most important variables for differentiation were size, effusion, age, and gender. PVNS had a size of 1–4 cm in a study by Murphey et al., although larger localized PVNS have been found [9, 10]. In our material all but one localized PVNS were ≤3 cm; that is, larger tumors should raise suspicion of a sarcoma. Importantly, however, intra-articular synovial sarcomas can be small [5]. Interestingly, moderate and large amounts of effusion were only found in PVNS. According to a study by Huang et al. on PVNS 8 out of 21 had effusion [10]. In previous reports of intra-articular synovial sarcomas a mean age of approximately 34 years has been presented, with only one patient being >50 years old [5]. The oldest sarcoma patient in our data was 35 years old. According to Murphey et al. PVNS is most common in the 3rd to 5th decades of life [9]. Since the male : female ratio is 1 : 1 in extra-articular synovial sarcomas, it was surprising that 6/7 intra-articular sarcoma patients were males. Friedman et al. have presented similar gender data on intra-articular synovial sarcomas with more than 70% of patients being males [5, 19]. However, the reason remains unclear. In synovial sarcomas chromosome X involves a* SYT-SSX* fusion gene, a very specific chromosomal translocation between chromosome X and chromosome 18 (t(X; 18)), which thereby could be subject to some gender difference [14].

Calcification and blooming were excluded in the multivariate data analysis due to several cases with missing radiographs or GRE sequences. Nevertheless, calcification is an important variable. Approximately 30% of synovial sarcomas have calcifications, while calcifications are not present in PVNS [6, 9]. In our material only 2 sarcomas had radiographs performed, but both had calcifications. This emphasizes the value of radiographs for differentiating intra-articular tumors. In our material only 7 patients had a GRE sequence performed, with 1/5 localized PVNS having “blooming” and 1/2 sarcomas also having “blooming.” Thus, it could be questioned if GRE sequence is of value in localized intra-articular tumors. Extra-articular growth was not found to be significant in our study. However, it was only seen in one case (Figure 2), which was a sarcoma. In this study we also evaluated low signal intensity in the synovia (suggesting hemosiderin) and synovitis (contrast enhancement and/or “rice bodies”), but none of them was significant. For the following variables the results were identical for both groups, well-defined border, bowl of grapes sign, triple signal intensity sign, and contrast enhancement, which therefore ought to be of no clinical use. Although evaluated in other studies, we choose not to test for lobulation or bone invasion. In our experience, lobulation is too subjective for evaluation, and without radiographs or computed tomography bone invasion is difficult to differentiate from pressure erosions, which can be seen in PVNS [8].

No sarcoma was misclassified as PVNS in the multivariate data analysis, while some PVNS were misclassified as sarcomas. Since sensitivity is more important than specificity for malignant tumors, this is useful in a clinical setting. A biopsy would be indicated when the multivariate data analysis suggests a sarcoma, while a biopsy could be avoided when the tumor is classified as most likely being a localized PVNS. However, one sarcoma was close to being misclassified as a PVNS. In clinical practice, when an unknown intra-articular tumor needs to be differentiated between a sarcoma and localized PVNS, it should be tested against the model and inserted to the PLS-DA *t*_1_ score plot. The further it is from the zero line, the stronger the suggestion is for either sarcoma or PVNS. This information will be regarded as additive to the radiologist's own judgement.

Extra-articular synovial sarcomas in the SSG Register had a higher mean age and larger mean size at diagnosis than intra-articular synovial sarcomas. This could be due to intra-articular tumors presenting with earlier symptoms. The incidence of synovial sarcoma arising in a joint has been unknown but believed to be low [6]. According to our data the incidence of intra-articular synovial sarcomas is 3% of all synovial sarcomas. The overall disease-free survival for intra-articular synovial sarcomas was 86% and for all synovial sarcomas 54%. For all synovial sarcomas in the SSG Register the disease-free survival was 98% in patients with tumor size ≤ 5 cm and age ≤ 20 years, compared to 32% in the group with size > 5 cm and age > 20 years. The only patient with intra-articular sarcoma in our study that did not survive had a tumor size of 3 cm.

There are some limitations to this study. Because of the rareness of the tumor, the material is small and the statistical analysis must be interpreted with care. However, we believe that the multivariate data analysis gives valuable clinical guidance. The database used only exists in our Sarcoma Center at this point, although an international database could be created. An advantage of using and expanding the database is that this could further improve the PLS-DA model in separating the tumors. We chose to include only localized PVNS as differential diagnosis, since other diagnoses rarely cause a problem. Multifocal PVNS is easily differentiated from a sarcoma, while hemangiomas and synovial osteochondromatosis also have a specific appearance [5, 6, 11]. Other sarcomas can exist intra-articularly but are even rarer. In 7 cases of localized PVNS there was no histopathological specimen, but all these patients are followed up at the Sarcoma Center with no suspicion of a sarcoma. In localized PVNS with no symptoms an operation is not advocated.

In conclusion, size, effusion, age, gender, and calcification are most useful for differentiation between sarcomas and localized PVNS. Sarcomas tend to be larger, have a small effusion, and be calcified, and the patients are younger and of male gender. To detect calcifications we recommend using radiographs as a complement to MRI. Although univariate analysis can be helpful, it is problematic in knowing how to best combine variables or which ones to rely upon. To overcome this a new approach was used, multivariate data analysis, which can be used as additive information to the radiologist. A biopsy could be avoided if it predicts that the tumor most likely is a localized PVNS.

## Figures and Tables

**Table 1:** Data on all synovial sarcomas from the SSG Central Register.

	Mean (SD)
Age (years)	38.8 (19.2)
Size (cm)	7.3 (4.9)

Male : female ratio	1 : 1
Metastasis at primary diagnosis	8.3%

Disease-free survival at latest follow-up	54%

Disease-free survival versus age^*∗*^	
≤20 years of age (68/83)	82%
>20 years of age (157/331)	47%

Disease-free survival versus tumor size^*∗∗*^	
≤5 cm (131/175)	75%
>5 cm (95/240)	40%

Disease-free survival versus age and tumor size	
≤20 years of age and ≤5 cm (40/41)	98%
>20 years of age and >5 cm (58/179)	32%

Patients with metastasis at primary diagnosis	Mean (SD)

Size (cm)	10.6 (4.8)

^*∗*^32 cases had missing data regarding age. ^*∗∗*^31 cases had missing data regarding tumor size.

**Table 2:** Univariate data analysis.

	PVNS	Sarcoma	*p* value
	Mean (range)	Mean (range)	
Size (cm)	2.5 (1.5–5)	4.6 (2–9)	0.059
Age (years)	41 (15–70)	21 (9–35)	0.012^*∗*^

	Number of patients/total#	Number of patients/total#	

Gender (male)	5/14	6/7	0.063
Extra-articular growth	0/14	1/7	0.33
Blooming	1/5	1/2	1.0
Calcification	0/7	2/2	0.028^*∗*^
Effusion			0.016^*∗*^
Small	6/14	7/7	
Moderate	5/14	0/7	
Large	3/14	0/7	
Low signal intensity in synovia	2/14	0/7	0.53
Synovitis	4/14	0/7	0.25

Total# refers to all patients with available information. ^*∗*^Significant prior to Bonferroni correction.

**Table 3:** Variable influence on projection (VIP) parameters for the variables in the model.

Age	1.32
Effusion	1.32
Size	1.22
Gender (F)	1.16
Gender (M)	1.16
Synovitis (Y)	0.84
Synovitis (N)	0.84
Extra-articular growth (Y)	0.78
Extra-articular growth (N)	0.78
Low signal intensity in synovia (Y)	0.56
Low signal intensity in synovia (N)	0.56

## References

[1] Walker E. A., Fenton M. E., Salesky J. S., Murphey M. D. (2011). Magnetic resonance imaging of benign soft tissue neoplasms in adults. *Radiologic Clinics of North America*.

[2] ESMO/European Sarcoma Network Working Group (2014). Soft tissue and visceral sarcomas: ESMO Clinical Practice Guidelines for diagnosis, treatment and follow-up. *Annals of Oncology*.

[3] Einarsdottir H., Söderlund V., Larson O., Jenner G., Bauer H. C. F. (1999). MR imaging of lipoma and liposarcoma. *Acta Radiologica*.

[4] Murphey M. D., Carroll J. F., Flemming D. J., Pope T. L., Gannon F. H., Kransdorf M. J. (2004). From the archives of the AFIP: benign musculoskeletal lipomatous lesions. *Radiographics*.

[5] Friedman M. V., Kyriakos M., Matava M. J., McDonald D. J., Jennings J. W., Wessell D. E. (2013). Intra-articular synovial sarcoma. *Skeletal Radiology*.

[6] Bui-Mansfield L. T., O'Brien S. D. (2008). Magnetic resonance appearance of intra-articular synovial sarcoma: case reports and review of the literature. *Journal of Computer Assisted Tomography*.

[7] Blacksin M. F., Siegel J. R., Benevenia J., Aisner S. C. (1997). Synovial sarcoma: frequency of nonaggressive MR characteristics. *Journal of Computer Assisted Tomography*.

[8] Murphey M. D., Gibson M. S., Jennings B. T., Crespo-Rodríguez A. M., Fanburg-Smith J., Gajewski D. A. (2006). Imaging of synovial sarcoma with radiologic-pathologic correlation. *Radiographics*.

[9] Murphey M. D., Rhee J. H., Lewis R. B., Fanburg-Smith J. C., Flemming D. J., Walker E. A. (2009). From the archives of the AFIP pigmented villonodular synovitis: radiologic-pathologic correlation. *Radiographics*.

[10] Huang G.-S., Lee C.-H., Chan W. P., Chen C.-Y., Yu J. S., Resnick D. (2003). Localized nodular synovitis of the knee: MR imaging appearance and clinical correlates in 21 patients. *American Journal of Roentgenology*.

[11] Helpert C., Davies A. M., Evans N., Grimer R. J. (2004). Differential diagnosis of tumours and tumour-like lesions of the infrapatellar (Hoffa's) fat pad: pictorial review with an emphasis on MR imaging. *European Radiology*.

[12] Gresswell S. D., Corsini A. A., Balsamo L. H., Miles E. F. (2013). Intra-articular synovial sarcoma treated with a transfemoral amputation: a case report and review of the literature. *Military Medicine*.

[13] Ishida T., Iijima T., Moriyama S., Nakamura C., Kitagawa T., Machinami R. (1996). Intra-articular calcifying synovial sarcoma mimicking synovial chondromatosis. *Skeletal Radiology*.

[14] Kimura H., Yamamoto N., Nishida H. (2014). Synovial sarcoma in knee joint, mimicking low-grade sarcoma confirmed by molecular detection of SYT gene split. *Anticancer Research*.

[15] Mann H. A., Hilton A., Goddard N. J., Smith M. A., Holloway B., Lee C. A. (2006). Synovial sarcoma mimicking haemophilic pseudotumour. *Sarcoma*.

[16] Namba Y., Kawai A., Naito N., Morimoto Y., Hanakawa S., Inoue H. (2002). Intraarticular synovial sarcoma confirmed by SYT-SSX fusion transcript. *Clinical Orthopaedics and Related Research*.

[17] Daniel A., Ullah E., Wahab S., Kumar V. (2009). Relevance of MRI in prediction of malignancy of musculoskeletal system—a prospective evaluation. *BMC Musculoskeletal Disorders*.

[18] Jones B. C., Sundaram M., Kransdorf M. J. (1993). Synovial sarcoma: MR imaging findings in 34 patients. *American Journal of Roentgenology*.

[19] Kransdorf M. J. (1995). Malignant soft-tissue tumors in a large referral population: distribution of diagnoses by age, sex, and location. *American Journal of Roentgenology*.

